# Focal adhesion kinase priming in pancreatic cancer, altering biomechanics to improve chemotherapy

**DOI:** 10.1042/BST20220162

**Published:** 2022-08-05

**Authors:** Kendelle J. Murphy, Jessie Zhu, Michael Trpceski, Brooke A. Pereira, Paul Timpson, David Herrmann

**Affiliations:** 1 Garvan Institute of Medical Research and The Kinghorn Cancer Centre, Darlinghurst, NSW 2010, Australia; 2 St Vincent's Clinical School, Faculty of Medicine, University of New South Wales, Sydney, NSW 2010 Australia

**Keywords:** biomechanics, FAK, fibrosis, pancreatic cancer, priming

## Abstract

The dense desmoplastic and fibrotic stroma is a characteristic feature of pancreatic ductal adenocarcinoma (PDAC), regulating disease progression, metastasis and response to treatment. Reciprocal interactions between the tumour and stroma are mediated by bidirectional integrin-mediated signalling, in particular by Focal Adhesion Kinase (FAK). FAK is often hyperactivated and overexpressed in aggressive cancers, promoting stromal remodelling and inducing tissue stiffness which can accelerate cancer cell proliferation, survival and chemoresistance. Therapeutic targeting of the PDAC stroma is an evolving area of interest for pre-clinical and clinical research, where a subtle reshaping of the stromal architecture prior to chemotherapy may prove promising in the clinical management of disease and overall patient survival. Here, we describe how transient stromal manipulation (or ‘priming’) via short-term FAK inhibition, rather than chronic treatment, can render PDAC cells exquisitely vulnerable to subsequent standard-of-care chemotherapy. We assess how our priming publication fits with the recent literature and describe in this perspective how this could impact future cancer treatment. This highlights the significance of treatment timing and warrants further consideration of anti-fibrotic therapies in the clinical management of PDAC and other fibrotic diseases.

## Introduction

Pancreatic cancer (PC) is predicted to be the second leading cause of cancer-related mortalities by 2026 [1]. Due to a lack of clinical features and perceptible symptoms at early-stage disease, a substantial proportion of patients present with inoperable locally advanced or metastatic cancer at diagnosis. Treatment options are often limited to systemic standard-of-care chemotherapy in PC patients. Despite the addition of nab-paclitaxel (Abraxane) to traditional gemcitabine monotherapy, and the recent introduction of Folfirinox into clinical practice, for a subset of relatively ‘fit’ PC patients, the average 5-year survival rate has remained largely unchanged over the last four decades at ∼11%, which is further decreased to 3% in the metastatic disease setting [2].

Originating in the exocrine cells of the pancreas, pancreatic ductal adenocarcinoma (PDAC), is the most commonly diagnosed type of PC, accounting for over 96% of all cases [3,4]. PDAC initiation and progression are accompanied by an enhanced fibrotic response, which involves extensive deposition and remodelling of the extracellular matrix (ECM [5–10]). This desmoplastic reaction is heterogeneous throughout the tumour and alters tissue stiffness in a spatially distinct manner which can also influence tumour cell dissemination [11–13]. Recent studies in PDAC and other cancers have shown that standard-of-care chemotherapy can have unintentional side effects on the stroma, for example, by triggering a chemotherapy-induced wound or fibrotic reaction leading to enhanced fibrosis and tumour stiffness, which is thought to further protect cancer cells from treatment [5,6,14,15]. This can lead to a vicious cycle of increased fibrosis and decreased response to standard-of-care chemotherapy [5,16]. Although ECM remodelling was previously thought to be predominantly mediated by cancer-associated fibroblasts (CAFs), recent studies suggest that both CAFs and cancer cells mediate tissue tension through the activation of mechano-signalling pathways such as; the integrin-Src-FAK signalling axis, JAK/STAT signalling and Rho kinases, which are all known to regulate cancer progression, metastasis and chemoresistance [9,17–22]. Despite the significant influence of the stroma on PDAC progression, several studies have demonstrated that the stroma can also restrain PDAC and that complete ablation of the stroma is accompanied by enhanced disease progression and metastatic capacity [23–27]. More recent studies however have shown that a transient manipulation of the ECM architecture and mechano-plasticity can prove promising by targeting the pro-tumorigenic functions of the stroma while enhancing chemotherapeutic efficiency [5,28–30]. Here, we discuss the significance of anti-fibrotic priming regimens targeting FAK and other molecules regulating cancer fibrosis, where balancing fine-tuned manipulation rather than long-term chronic treatment may prove beneficial in the clinical setting.

## FAK protein structure and role in cancer

FAK is a ubiquitously expressed adaptor protein and non-receptor tyrosine kinase. As a major signalling hub integrating extracellular and intracellular stimuli, FAK mediates bidirectional integrin-mediated cell adhesion and migration signals [31–34]. FAK is composed of an N-terminal 4.1 ezrin radixin moesin (FERM) homology domain and a C-terminal focal adhesion targeting (FAT) domain which flank a central kinase domain. The N- and C-termini are separated from the kinase domain by linker regions which contain the proline-rich regions 1, 2 and 3. Important Tyrosine phosphorylation sites along FAK include Tyr-397, which has a crucial role in FAK activation. The autophosphorylation of Y397 is a key step in the activation of FAK signalling and as such this catalytic site is a major target of therapeutic agents [35]. Furthermore, the adaptor functions of FAK, specifically those at the cell-ECM interface influencing focal adhesion turnover have been well recognised [32,36]. FAK is often overexpressed and hyperactivated in multiple aggressive cancers such as pancreatic cancer, mesothelioma and ovarian cancer and correlates with poor survival [5,32,35,37,38]. The reciprocal tumour-stromal interactions mediated by FAK drive pathways known to modulate matrix architecture and tissue stiffness as well as cell proliferation, survival and disease progression [17,37,39]. As such, FAK inhibitors have promising utility in the clinical management of multiple cancer types including PDAC.

## FAK priming in PDAC; a dual-targeting approach

The well-known Y397 autophosphorylation site in FAK has led to the development and clinical assessment of numerous FAK inhibitors including VS-6063 (defactinib), VS-4718, IN10018 (formerly BI 853520), PF-04554878, PF-562271, GSK2256098, PF-573228 and recently AMP945 [32,37,40–49]. To date, clinical assessment of single-agent FAK inhibition has resulted in disease stabilisation in solid tumours including colorectal, ovarian and pancreatic cancer [42,50] with the potential of FAK inhibitors to enhance treatment efficacy in combination therapies warranting further investigation. Recent studies suggest that targeting FAK will be most effective in combination with standard-of-care chemotherapy agents or immunotherapy to enhance their efficacy or to overcome adaptive treatment resistance [30,46,51–56]. In this manner, our recent publication assessed the efficiency of short-term FAK priming prior to chemotherapy to render cells vulnerable to subsequent chemotherapy, reduce metastatic spread and impair disease progression [5].

Validated primary PDAC cells isolated from the highly metastatic KPC mouse model and patient-derived cell lines (PDCLs) from the Australian Pancreatic Genome Initiative (APGI) served as pre-clinical models to streamline the benefits of FAK priming in murine and human PDAC tissue [5]. We demonstrated that early pre-treatment (or ‘priming’) with a FAK inhibitor (FAKi) for 3 days prior to chemotherapy reduced stromal fibrosis and stiffness and caused an increase in the proportion of PDAC cells in the G2/M phase of the cell cycle, rendering them exquisitely vulnerable to gemcitabine and Abraxane chemotherapy. Critically, short-term priming was better or equal to chronic treatment highlighting the pivotal role that the timing and fine-tuning of targeting the tumour microenvironment (TME) can play in cancer outcomes.

**Figure 1.**
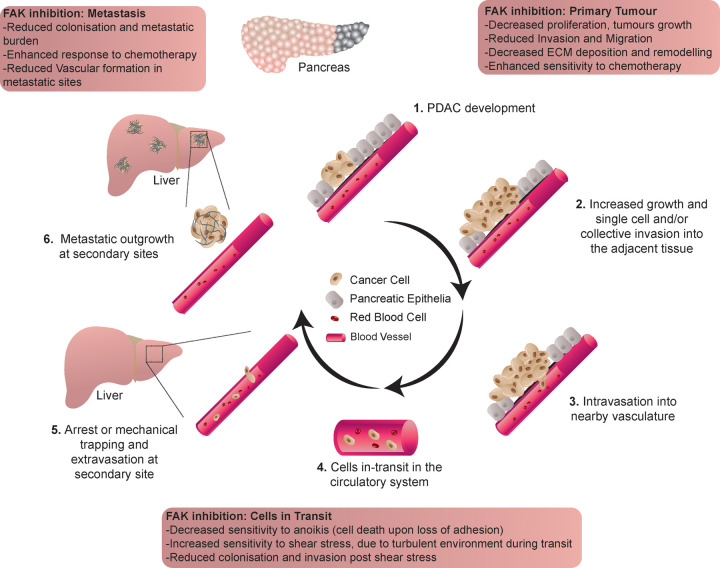
Schematic representation of the metastatic cascade, and the effect of FAK inhibition. Specifically highlighting **1.** PDAC development and the acquisition of a motile phenotype, **2.** Increased growth and invasion into adjacent tissue, followed by **3.** Intravasation in nearby vasculature. Once in the vasculature **4.** Cells transit in the circulatory system until **5.** Arrest or mechanical trapping and extravasation with subsequent **6.** Metastatic outgrowth at secondary sites such as the liver.

In line with this, intravital (*in vivo*) imaging can provide real-time insight into how cells behave in their native microenvironment, including during cell invasion and migration and following drug treatment influenced by local environmental cues, such as fibrotic ECM and adjacent vasculature. Importantly, we used intravital imaging to observe and track PDAC response to anti-fibrotic FAKi treatment in combination with chemotherapy allowing us to optimise treatment regimens in live PDAC tissue [5]. In particular, using the FAK-based Förster resonance energy transfer (FRET) biosensor [57] and the Fluorescent Ubiquitination-based Cell Cycle Indicator (FUCCI) cell cycle reporter [58], we monitored dynamic changes in FAK signalling and cell cycle progression, respectively, in primary and secondary PDAC sites. First, we showed a decrease in FAK activity upon FAKi treatment, as demonstrated by intravital imaging of the FAK-FRET biosensor in live primary PDAC tumours. Transient FAK priming also led to a subtle change in the ultrastructure of the ECM, quantified via Second Harmonic Generation (SHG) imaging of collagen fibres. Lastly, analysis of the FUCCI cell cycle reporter revealed an accumulation of PDAC cells residing in the G2/M phase upon FAKi priming followed by gemcitabine/Abraxane chemotherapy in live primary tumours and secondary liver metastases (Figure 1), which may indicate effective PDAC cell cycle stalling or arrest at both sites.

To further assess the mechanism of cancer cell chemo-sensitisation following FAKi priming, we aimed to uncouple the direct effect of FAK inhibition and the contribution of altered stromal biomechanics caused by FAKi priming. We utilised micropatterned pillar plates and stiffness-tuneable matrices to recapitulate changes in matrix stiffness following FAKi priming [5]. Here, quantification of cell cycle distribution using the FUCCI reporter revealed that reduced matrix stiffness rather than direct epithelial FAK inhibition dictates PDAC cell chemosensitivity by increasing the number of PDAC cells in the G2/M phase. Encouragingly, similar studies in PDAC showed that FAK inhibition synergises with Abraxane to reduce cell proliferation *in vitro* and decrease tumour growth *in vivo* [59]*,* further highlighting the potential of FAK dual-targeting with standard-of-care chemotherapy. This is in line with recent studies from our laboratory and others, where similar short-term pulsed treatment schedules using the ROCK inhibitor Fasudil, led to decreased stromal fibrosis and increased vascular patency, thus improving chemotherapy delivery and performance [19,28].

PDAC is a highly metastatic malignancy, with most patients presenting to the clinic with disease spread to other organs at initial diagnosis [2]. The multistep process of metastasis, known as the metastatic cascade, can initially involve the acquisition of a mobile cancer cell phenotype and localised invasion (Figure 1.1 and 1.2, [60–63]). Using cell-derived matrices (CDMs) and organotypic invasion assays, we assessed the potential of stromal FAKi priming to subsequently impair the migratory and invasive capacity of PDAC cells (Figure 1.2, [64,65]). Here, treatment of fibroblasts with FAKi during CDM production significantly reduced the deposition and altered the architectural composition of the matrix. Interestingly, on CDMs primed with FAKi, collective cell migration was impaired, with cells moving in an uncoordinated manner (Figure 1.2, [5]). Similarly, early FAKi priming of organotypic matrices inhibited fibroblast-mediated contraction, leading to a disruption of the collagen ultrastructure and reduced matrix stiffness. These stromal alterations upon early FAKi priming efficiently impaired cancer cell invasion (Figure 1.2) and improved subsequent chemotherapy performance thus highlighting the efficiency of staggered combination therapy regimens, which can reduce the need for chronic treatment schedules [5].

Following cell movement and detachment from the primary tumour, cells can intravasate into neighbouring vascular or lymphatic vessels that provide a systemic transport route to distant organs (Figure 1.3 [66]). Cells in transit through the vascular system are subject to a range of detrimental factors including the loss of cellular adhesion and physical forces, such as fluid flow-induced shear stress [67]. To successfully extravasate and colonise secondary sites, malignant cells have to adapt to survive the harsh vascular environment [66,68]. To mimic the loss of cellular adhesion and physical forces during vascular transit, we exposed PDAC cells *in vitro* to anchorage-independent growth (AIG) and a controlled physiologically relevant level of shear stress (Figure 1.4). Here, AIG assays showed that FAKi alone or prior to chemotherapy reduced cell growth in anchorage-independent conditions [5]. Furthermore, shear stress assays revealed that FAKi priming increased cell apoptosis whilst also reducing subsequent cell invasion into organotypic matrices, as a surrogate readout of cell movement and colonisation at secondary sites (Figure 1.5). To further corroborate these findings *in vivo*, we assessed the effect of FAKi priming prior to chemotherapy on liver colonisation upon intrasplenic injection of PDAC cells (Figure 1.6). This study revealed that FAKi alone or in combination with chemotherapy impaired the ability of PDAC cells in transit to extravasate and colonise the liver *in vivo* [5]. Moreover, histological analysis revealed that FAKi may also disrupt the formation of nascent blood vessels in the liver, which could further inhibit metastatic establishment and aligns with the known role of FAK in angiogenesis [69,70]. This is in line with a recent study which found that loss of endothelial FAK in mouse models of PDAC did not alter primary tumour growth but significantly reduced liver metastatic burden, and improved response to gemcitabine monotherapy [71]. Moreover, low levels of endothelial FAK were found to correlate with increased survival and reduced likelihood of relapse in PDAC patients upon gemcitabine treatment [71]. Collectively, these results demonstrate that FAK regulates several distinct aspects of the metastatic cascade in PDAC, in both cancer and stromal cells at the secondary site, highlighting the influence of the liver metastatic niche (Figure 1 [72]). Furthermore, this may also partially explain the anti-metastatic efficacy of FAKi alone and followed by chemotherapy we observed *in vivo* [5].

PDAC is a highly heterogeneous disease, where intratumoral and intertumoral variability means that different patients may require different therapeutic approaches [73–75]. In line with this, our study has revealed a graded response to FAKi priming in patient-derived xenograft (PDX) tissue from different patients. We observed a significant extension in the time to PDAC metastasis and overall survival upon FAKi priming followed by chemotherapy in a PDX model with high FAK activity with only 50% of mice exhibiting overt metastasis at study endpoint. In contrast, a PDX model with low FAK activity only showed a significant extension in time to PDAC metastasis upon combination therapy without changes in overall survival [5]. This graded response highlights the need to stratify PDAC patients according to their individual tumours’ molecular signature and to identify additional companion biomarkers to guide clinical intervention in a personalised medicine approach. Previous studies in mesothelioma and ovarian cancer revealed that Merlin expression may be associated with sensitivity to FAK inhibitors and that low Merlin/high FAK co-correlate with poor patient survival in pancreatic cancer [5,37,38,42,76].

Merlin also known as NF2 functions as a linker between transmembrane proteins and the actin cytoskeleton. In cells with high Merlin expression, stable cell–cell junctions decrease cell dependence on cell-ECM adhesion, which is largely mediated by FAK signalling [77]. Conversely, cells with low Merlin expression rely more on ECM contacts for survival thus enhancing their sensitivity to FAK inhibitors. We assessed our PDX models for Merlin expression and identified low Merlin expression in the responder model compared with high Merlin expression in the non-responding PDX model. Interestingly, genetically decreasing Merlin expression in the PDX model, which was previously non-responsive to FAKi priming prior to chemotherapy, now rendered this PDX vulnerable to our combination therapy resulting in disease stabilisation [5]. This indicates that Merlin could also be used as a companion biomarker in PDAC to predict whether FAKi priming followed by chemotherapy may be beneficial to improve survival in a subset of patients, and thus warrants further clinical assessment.

## Other strategies to target FAK in pancreatic and other cancers

Since its identification as a driver of tumour progression and malignancy several FAK inhibitors have been explored within the clinic (please also see Table 1 for a non-exhaustive summary of clinical approaches to target FAK and other molecules regulating the TME). The ATP-competitive inhibitor of FAK, PF-573228, was first developed by Pfizer and has served as a prototype for newer generations of FAK inhibitors [41]. Following promising pre-clinical studies, the FAK inhibitor PF-562271 was the first FAK inhibitor to enter clinical trials in solid tumours including, pancreatic, head and neck, and prostate cancer (NCT00666926), with one-third of patients showing disease stabilisation [34,78,79]. Defactinib (VS-6063) exhibits increased specificity towards FAK and superior pharmacodynamics compared with PF-562271, which was previously shown to also inhibit CYP3A, a major metabolising enzyme of cytotoxic agents [78,80]. As such Defactinib can offer a safer less toxic alternative when combining FAK inhibition with chemotherapy. An initial phase II clinical trial in lung carcinoma patients with mutant Kras showed that Defactinib was well tolerated and yielded a modest response in terms of survival and tumour progression (NCT01951690, Table 1, [45]). Furthermore, interim data from a phase I/II trial of Defactinib in combination with the chemotherapy paclitaxel in ovarian cancer (NCT01778803) has also revealed promising results, with 64% of patients showing disease stabilisation (Table 1, [81]). This highlights the need to further assess how we can best combine FAKi treatments with chemotherapy to improve upon these promising clinical results.

**Table 1:** Clinical Trials Assessing FAK inhibition and other treatment strategies to modulate the TME

Target	Drug	Combination	Patients	Phase	Status	NIH number
FAK	Defactinib	PD-1	Mesothelioma, NSCLC, PC	I/II	Recruiting	NCT02758587
	Defactinib	PD-1, gemcitabine, nab-paclitaxel	Solid tumours, PC	I /II	Recruiting	NCT02546531
	GSK2256098	Trametinib	PDAC	II	Recruiting	NCT02428270
	Sonidegib phosphate		Basal cell carcinoma (advanced and metastatic)	II	Ongoing	NCT013270053 (BOLT)
	VS-6766	Defactinib	NSCLC, Low-Grade Serous Ovarian Cancer, Endometrioid Cancer, Pancreatic Cancer	I	Recruiting	NCT03875820 (FRAME)
	VS-6766 vs VS-6766 + Defactinib	Vehicle or Defactinib	Ovarian Cancer (low grade with or without KRAS mutation)	II	Recruiting	NCT04625270 (RAMP-201)
	VS-6766 vs VS-6766 + Defactinib	Vehicle or Defactinib	NSCLC (KRAS G12V, or other KRAS or BRAF mutations)	II	Recruiting	NCT04620330 (RAMP-202)
	Defactinib		KRAS Mutant NSCLE	II	Completed	NCT01951690
	Defactinib	Stereotactic Body Radiotherapy	PDAC	II	Recruiting	NCT04331041
	Defactinib	Paclitaxel	Advanced Ovarian Cancer	I	Completed	NCT01778803
All-trans-retinoic acid		Gemcitabine, nab-paclitaxel	PDAC	I	Completed, no data released	NCT03307148 (STARPAC)
Galectin-3	GB1211	Atezolizumab	NSCLC	I/II	Recruiting	NCT05240131
Bruton's tyrosine kinase (BTK)	Ibrutinib	Gemcitabine, nab-paclitaxel	Metastatic PDAC	III	Completed	NCT02436668 (REVOLVE)
Hyaluronan	pegvorhyaluronidase alfa (PEGPH20)		Metastatic PDAC: assessment of blood-based biomarkers as predictors of survival	II	Completed	NCT01839487 (HALO109-202)
	PEGPH20	Pembrolizumab	Solid Tumours (NSCLC, Gastric Cancer): selected based on High Hyaluronan status	I	Completed	NCT02563548
Fibrosis	Pirfenidone	Standard first-line chemotherapy (Carboplatin, Paclitaxel, Pemetrexed)	Advanced-Stage Lung NSCLC	I	Active, not recruiting	NCT03177291
HGF	YYB101		Solid Tumours	I	Completed	NCT02499224
	Pamrevlumab	Substudy: Pirfenidone, Nintedanib	Idiopathic Pulmonary Fibrosis	II	Completed	NCT01890265

In pancreatic cancer, FAK hyperactivity is known to be a regulator of an immunosuppressive TME which drives disease progression [30]. Initial studies in squamous cell carcinoma (SCC) on the connection between FAK and the immune system showed that nuclear-targeted FAK promotes tumour immune evasion, due to exhaustion of CD8+ T cells and the recruitment of pro-tumourigenic regulatory T cells (Tregs), driving cancer progression. This tumour-promoting effect of nuclear FAK was reversed using VS-4718 which drove the depletion of Tregs and promoted a CD8+ T cell-mediated anti-tumour response [82]. Additionally, nuclear FAK led to an enrichment in the expression of genes that encode for interleukin-33 (IL-33) and the chemokine CCL5, as well as the secretion of the soluble form of the IL-33 receptor; soluble ST2 (ST2) [83]. Here, the nuclear FAK-IL-33 complex interacted with transcriptional regulators and chromatin modifiers to promote NF-κB activity, which induced chemokine expression, including CCL5. The over-production and secretion of ST2 are thought to sequester IL-33 present within the TME, blocking its stimulation of infiltrating immune cells and driving tumour progression [83]. Further studies assessing the modulation of the immune system and FAK by Jiang et al. [30]. found that FAK activity was elevated in human PDAC, in correlation with poor CD8+ T cell infiltration and fibrotic disease, and that high FAK activity and low CD8+ levels were predictive of poor survival Here, FAK inhibition in combination with gemcitabine and adoptive T cell transfer or immune checkpoint inhibitors was shown to increase sensitivity to chemo- and immunotherapy and to double PDAC survival in mouse models [30]. This study led to a phase I clinical trial assessing the combination of the FAKi VS-6063 with PD-1 antagonist immunotherapy alone or in combination with gemcitabine and showed a positive response with 53% of patients presenting with stable disease in the triple combination [84]. Similarly, high-grade serous ovarian carcinoma displays resistance to immunotherapy, due to the elevated expression of immune checkpoint ligands which restricts anti-tumour immunosurveillance [85]. Multiplexed analysis of tumour cells showed that FAK regulates the expression of CD155, a checkpoint ligand for TIGIT (T cell immunoreceptor with immunoglobulin and immunoreceptor tyrosine-based inhibitory motif domains) [85]. Here, FAK inhibition via VS-4718 combined with blocking TIGIT antibody immunotherapy led to immune cell activation and decreased tumour burden, enhancing mouse survival [85].

In PDAC, IN10018, a small molecule inhibitor of FAK, was shown to enhance the anti-tumour efficacy of radiotherapy, which resulted in reduced tumour growth and increased survival [49]. Further investigation revealed that this effect was due to an increase in anti-tumorigenic CD8+ T cells and an inhibition of infiltrating pro-tumourigenic granulocytes [49]. Similarly, a recent study using antibody-driven expression of the T cell stimulatory ligands CD80, 4-1bb and OX40 sensitised SCC and PC cells to FAK inhibition in combination with gemcitabine [86]. Despite these promising pre-clinical and clinical results, continuous treatment with FAK inhibitors can lead to PDAC resistance to FAKi therapy. For example, chronic treatment of PDAC-bearing mice with VS-4718, (a bispecific inhibitor of FAK and Pyk2 kinases), was shown to reprogram the TME which induced a feedback loop and decreased stromal TGF-β along with hyperactivated STAT3 signalling. This in turn enhanced cell proliferation and led to eventual treatment resistance. This resistance to FAKi could be overcome by combination treatment with JNK/STAT3 and FAK inhibitors, showing durable efficacy in PDAC models [87].

In addition to cancer cells’ intrinsic abilities to evade treatment and reinforce extrinsic signals, such as fibrosis, FAK can help provide a safe haven for cancer cells. Using intravital imaging of an ERK/MAPK biosensor, Hirata et al. [51] monitored the response of the TME to BRAF inhibition in melanoma. Despite initial response in areas of high stromal density, rapid ERK/MAPK reactivation was observed. This was linked to the activation of cancer-associated fibroblasts (CAFs), promoting ECM deposition and remodelling and enhancing integrin/FAK/Src signalling [51]. Here, dual treatment with a BRAF and a FAK inhibitor prevented ERK reactivation and led to more effective control of tumour growth [51]. Collectively, these studies highlight (i). the significance of targeting FAK in anti-cancer therapies, (ii). the versatility of FAK inhibitors to improve upon other treatment modalities and (iii). the role of treatment timing to maximise therapeutic efficacy whilst reducing the likelihood of drug toxicity and acquisition of treatment resistance, which are often associated with long-term, chronic therapies.

The potential of FAK targeting has also been well researched in other cancers. In breast cancer, the FAK inhibitor BI-853520 was shown to reduce cell proliferation resulting in a significant reduction in primary tumour growth [88]. Interestingly, in the metastatic setting, early treatment with BI-853520 3 days prior to tumour cell injection reduced the metastatic nodule size in the lung. However, delayed treatment with FAKi post tumour cell injection did not significantly reduce the number of metastases [88]. This suggests that BI-853520 is a potent inhibitor of early metastatic outgrowth, further highlighting the potential of FAKi as an early priming agent [88]. Another study in triple-negative breast cancer (TNBC), a highly invasive and metastatic molecular subtype of breast cancer, revealed that the mRNA expression levels of FAK are higher in TNBC than in tumours from non-TNBC subtypes and normal breast tissues [89]. Here, increased expression of FAK in TNBC was found to be driven by oestrogenic G protein-coupled oestrogen receptor (GPER) signalling. This led to STAT3 nuclear accumulation and increased TNBC cell invasiveness, which could be reduced by using the FAK inhibitor VS-4718 [89]. In another cohort of invasive TNBC patient samples, high FAK expression was shown to correlate with an increased risk of cancer recurrence and reduced patient survival [90]. Moreover, co-expression of FAK and markers of cancer stem cells (CSCs), which are known to contribute to therapy resistance and disease recurrence, was associated with poor prognosis [90]. In this context, FAK inhibition resulted in a decrease in CSC activity and cell proliferation, and the combination of the FAKi VS-4718 and paclitaxel further reduced TNBC CSC renewal, potentially improving long-term survival [90]. This indicates that combining FAK inhibition in combination with adjuvant therapy has the potential to improve survival in breast cancer patients.

## Blunting fibrosis and modulating the TME in cancer

The aggressive nature of PDAC is in part due to the distinct mechanical features of the ECM [10]. This has led to the re-purposing of several other anti-fibrotic agents in addition to FAK inhibitors for PDAC treatment. For example, the humanised monoclonal antibody, pamrevlumab, targets Connective Tissue Growth Factor (CTGF) and is presently used for the clinical management of idiopathic pulmonary fibrosis [91]. In combination with gemcitabine/Abraxane, pamrevlumab was recently assessed in a Phase I/II clinical trial for locally advanced PDAC, where it led to an increased proportion of patients becoming eligible for surgical resection compared with those who received only chemotherapy (NCT01890265, Table 1, [91,92]). This trial highlights the potential of anti-fibrotic targeting in neoadjuvant PDAC therapy to shrink tumours prior to surgery. Similarly, the clinically approved Bruton Tyrosine Kinase (BTK) inhibitor, Ibrutinib, has been shown to exert potent anti-fibrotic and anti-tumour activities in mouse models of PDAC [93]. *In vivo* studies showed that Ibrutinib reduced fibrosis by preventing the secretion of IL8, MPC-1 and TNF-α from mast cells [93], which are known to promote fibrotic disease [94,95]. Furthermore, the combination of Ibrutinib with gemcitabine improved chemotherapy efficacy, whilst also neutralising chemotherapy-induced toxicity, resulting in significantly extended survival compared with gemcitabine monotherapy [93]. This promising data has since moved to the clinic with a Phase III trial evaluating Ibrutinib in combination with gemcitabine/Abraxane as first-line treatment for metastatic PDAC (NCT02436668, Table 1, [96]).

To further understand the contribution of the TME to cancer progression, and identify druggable targets, recent studies have focused on the molecular profiling of both cancer cells and CAFs [9,75,97–101]. In various high-grade aggressive cancers, including pancreatic, lung, head and neck and breast cancers, RNA expression profiling showed that leucine-rich repeat 15 (LRRC15) is highly expressed on CAFs, and could be a novel CAF and mesenchymal stem cell marker [102]. LRRC15 is involved in the regulation of multiple biological processes including cell adhesion, invasion and survival as well as modulating the immune response, ECM assembly and RNA processing. In a model of non-small cell lung adenocarcinoma, ABBV-085 (anti-LRRC15; a monomethyl auristatin E containing antibody-drug conjugate against LRRC15) treatment in combination with gemcitabine showed enhanced anti-tumorigenic efficacy in solid tumours compared with single-agent therapies, suggesting that it enhanced the delivery and/or efficacy of chemotherapy [102].

Within PC, heterogeneous subtypes of CAFs co-exist that can both promote and restrain disease progression, influencing all aspects of the TME [97,101,103]. For example, CAFs were shown to regulate malignant cell metabolism through FAK-mediated activation of stromal signalling pathways [104]. Here, FAK depletion in CAF subpopulations regulated paracrine signals which increased chemokine production and subsequently enhanced glycolysis in cancer cells, driving tumour growth [104]. Future studies will shed further light on the role of FAK signalling in different CAF subtypes or assess how FAK inhibition might affect the abundance of different CAF subtypes in a tumour. Moreover, we recently revealed a p53-mediated hierarchy, whereby a gain-of-function mutation in p53 can educate a population of CAFs to establish a pro-metastatic environment [75]. Perlecan was identified as a key component of this pro-metastatic TME and depletion of Perlecan enhanced the efficacy of chemotherapy, prolonging survival in mouse models [75,99]. Similarly, disruption of tumour-stroma interactions via the hepatocyte growth factor (HGF)/c-MET pathway using a triple therapy combining the humanised monoclonal anti-HGF antibody AMG102, a c-MET inhibitor and gemcitabine chemotherapy led to significant inhibition of cell proliferation *in vitro* and reduced tumour burden and metastasis *in vivo* [105]. These findings led to a first-in-human phase I trial of the clinically relevant anti-HGF antibody, YYB101 in refractory solid tumours, revealing favourable safety and efficacy [106], and presenting a new therapeutic drug candidate for the treatment of PDAC patients (NCT02499224, Table 1).

## Future directions

Anti-fibrotic treatments designed to target unique aspects of the tumour microenvironment offer promising new clinical opportunities for improving patient survival in both PDAC and other highly fibrotic cancer types. Currently, several clinical trials targeting FAK or other molecules regulating fibrosis are underway in different cancers, to assess the potential of stromal targeting in combination with other treatments to enhance patient survival (Table 1). Here, patient stratification according to biomarkers, including FAK or Merlin, may help to identify, which patients are most likely to benefit from therapeutic intervention. Due to the numerous roles and functions of the stroma, anti-fibrotic therapies can provide multiple benefits including a reduction in primary tumour growth and cancer metastasis as well as improved response to chemotherapy and other common treatment modalities, which thus may further extend patient survival using combination therapies. In particular, transient or short-term manipulation of the stroma can deprive cancer cells of their supportive niche, whilst also minimising the likelihood of drug resistance and toxicity, which are often associated with long-term chronic treatments. In this context, intravital imaging and the live tracking of cancer cells can help optimise the efficacy of combination treatment schedules prior to long-term studies [107–109]. Beyond this, dual or companion biomarkers may facilitate the identification of patient subsets that are highly likely to respond to anti-fibrotic therapy in combination with other treatment approaches.

We recently showed that sequential treatment regimens consisting of short-term FAK inhibition prior to standard-of-care chemotherapy may provide a promising strategy to be assessed in PDAC patients. With the ongoing emergence of novel anti-fibrotic therapies and the re-purposing of existing anti-fibrotic agents for cancer treatment, the aspect of knowing how best to combine different therapies in a personalised manner to achieve maximum response in patients is becoming more and more relevant.

## Perspectives

- The dense fibrotic stroma of pancreatic cancer is known to promote cancer spread and impair the efficacy of standard-of-care chemotherapy. As such novel treatment regimens are needed to target the fibrotic stroma and improve overall survival.
- Whilst anti-fibrotic therapies, such as FAK inhibitors, can offer promising new clinical opportunities for pancreatic cancer management, it is not obvious how to combine them with other treatments to maximise therapeutic efficacy. Using intravital imaging we showed that short-term transient inhibition of FAK can deprive pancreatic cancer cells of their supportive fibrotic niche and render them exquisitely vulnerable to subsequent chemotherapy, whilst minimising the likelihood of resistance and toxicity occurring due to long-term chronic treatment.
- Future applications of anti-fibrotic therapies in combination with standard-of-care chemotherapy and other treatment modalities may focus on how best to combine different treatments to maximise treatment benefits. Moreover, dual or companion biomarker-driven studies may help to determine those patients who are likely to respond to combination treatments involving anti-fibrotic therapies.

## Abbreviations

AIG: anchorage-independent growth
BTK: Bruton Tyrosine Kinase
CAFs: cancer-associated fibroblasts
CDMs: cell-derived matrices
ECM: extracellular matrix
FAK: focal adhesion kinase
FRET: Förster resonance energy transfer
FUCCI: Fluorescent Ubiquitination-based Cell Cycle Indicator
HGF: hepatocyte growth factor
PC: pancreatic cancer
PDAC: pancreatic ductal adenocarcinoma
PDX: patient-derived xenograft
SCC: squamous cell carcinoma
TME: tumour microenvironment
TNBC: triple-negative breast cancer
